# Treatment of simple bone cysts of the humerus by intramedullary nailing and steroid injection

**DOI:** 10.1186/s12891-020-3054-6

**Published:** 2020-02-04

**Authors:** Peng Zhang, Naiqiang Zhu, Lilong Du, Jihui Zheng, Sibin Hu, Baoshan Xu

**Affiliations:** 1 Graduate School, Tianjin Medical University, Heping District, Tianjin, 300070 People’s Republic of China; 2 Department of Orthopedics, Tianjin Hospital, Jiefang South Road 406, Hexi district, Tianjin, 300211 People’s Republic of China; 3 Department of Orthopedics, Cangzhou Hospital of Integrated TCM-WM·Hebei, Cangzhou, Hebei 061000 People’s Republic of China

**Keywords:** Simple bone cyst of humerus, Children, ESIN, Steroids, Combination therapy

## Abstract

**Background:**

Simple bone cysts (SBCs) are common benign lytic bone lesions in children. This study focused on exploring a clinical treatment method, minimally invasive intramedullary decompression and drainage with elastic stable intramedullary nailing (ESIN) combined with intralesional injections of steroids, and evaluated its effectiveness, complications and morbidity through functional and radiographic outcomes.

**Methods:**

The postoperative recovery of 18 children who suffered from SBCs of humerus was evaluated (mean follow-up, 40 months) from January 2009 to December 2016. These patients (11 males, 7 females; 8 in the left, 10 in the right; mean age, 10.9 years old) were treated with minimally invasive intramedullary decompression and drainage with ESIN combined with intralesional injections of steroids. The diagnosis was based on not only pre-operative typical medical images (X-rays/CT/MRI) but also surgical findings and pathological diagnosis. Radiological and functional outcomes were evaluated according to Capanna and Musculoskeletal Tumor Society (MSTS) score. The interclass differences were analyzed by t-test.

**Results:**

According to Capanna and MSTS criteria, after treatment, 14 patients made full recoveries which was presented by all the cysts filled with bone tissue, and 4 patients made partially recoveries, which were presented by cystic spaces partially filled with low density bone. All the cysts responded to treatment method, and there was no cyst recurrence. All except 2 patients had good functional results. One of the two patients had irritation of the end of the nail and one patient had a valgus deformity.

**Conclusions:**

Treatment for SBCs of humerus by minimally invasive intramedullary decompression and drainage with ESIN combined with intralesional injections of steroids is safe, effective and convenient. The clinical effect is satisfactory and worth popularizing.

## Background

According to Virchow, simple bone cysts (SBCs), also called unicameral bone cysts (UBCs), are benign fluid-filled bone tumors, which commonly and typically locate in metaphysis of long bones in children [1]. SBCs are usually discovered in the presence of pathological fractures [2–4], and they are more common in long bones, especially in humerus and femur, and less common in tibia, fibula, radius, and ulna [5, 6]. The sex ratio of male to female patients with SBCs is approximately 2:1, indicating the incidence of this disease may be related to gender [7]. In addition, many studies have raised other relevant hypotheses on its pathogenesis, including venous obstruction, and destructive factors like interleukin (IL)-1 and Prostaglandin (PG) [8, 9]. There is no specific and standard therapeutic schedule in terms of the treatment of SBCs. The surgical methods commonly used in clinical practice are total resection with bone grafting to remove all the cyst and associate bone tissue, and subtotal resection with or without bone grafting [10, 11]. Those aggressive operations are extensive and complex, often with high complications [12]. In recent decades, new treatments have been developed, such as intralesional injections of steroids [13, 14], intralesional injections of bone marrow [15], bone grafting with homologous cancellous bone chips [16], bone grafting with freeze-dried crushed cortical bone [17], and decompression with screws or pins with holes [18]. Even though these new surgical treatments present promising short-term outcomes, most of them are likely to end up with partial recoveries [19], and the recrudesce and tenacity are still the biggest challenges [3].

Recently, with the rapid development of minimally invasive surgical techniques, great changes have taken place in the treatment of SBCs by percutaneous intramedullary decompression. Santori et al. was the first to report elastic nails in 1986 [20]. Then, elastic nails were used in the treatment of unicameral bone cyst in long bones by Roposch’s group [21]. When the elastic intramedullary nails were inserted between the medullary canal and the cyst cavity, continuous drainage and intracystic pressure decompression were carried out. Furthermore, elastic intramedullary nails could play a stable and supporting role, enabling early postoperative motion, preventing adjacent joint stiffness and promoting healing, particularly in the treatment of pathological fractures [22, 23]. However, it was reported that residual lesions were found in the patients with SBCs after treated of only by elastic stable intramedullary nailing (ESIN) [24, 25].

Various methods in the treatment of SBCs ended up with partial healing or residual lesions, thus there is a debate on whether to take conservative treatment or aggressive surgical treatment. However, as SBCs are typically located in metaphysis of long bones in juvenile children, who are eager to return to sports and activities, the minimally invasive and reliable fixed method seemed to be the optimal choice. To explore a better treatment, with a retrospective of SBCs’ etiology, we found both venous outflow obstruction and PG E2/IL-1β enzymes within the cyst fluid, which would cause bone destruction [26]. Considering the validity of minimally invasive intramedullary decompression, drainage with ESIN, and steroids intralesional injection to these pathogenic factors, and there were no related reports about a combined method before, we conducted the current research to assess the safety and effectiveness of this combination therapy in the clinical practice.

## Methods

### Clinical information

This retrospective study was approved by the local ethics committee. From January 2009 to December 2016, 18 children who had an SBC of the humerus were treated with intramedullary decompression and drainage with ESIN combined with intralesional injections of steroids. The surgery-indications of these patients included large and painful SBCs with or without pathological fracture. Diagnosis information was obtained from orthopedic files, including preoperative/postoperative X-rays, computed tomography (CT) and magnetic resonance imaging (MRI). Clinical data included gender, age, symptoms, presence or absence of pathological fracture, surgical procedures, and functional or radiological outcomes.

As well known, X-ray images of SBCs show that the medullary cavity is a central elliptical bright shadow with no gravel-like densification point inside, which sometimes is segregated by bone ridges, and cortical bone will expand and becomes thinner, but there is no periosteal reaction (except pathological bones). Magnetic Resonance Imaging (MRI) always presents a low or intermediate signal on T1-weighted images and a homogeneous high signal on T2 weighting. The cystic fluids extracted by surgeon are tested for pathological examination, which can confirm the diagnosis of SBCs. In this study, considering the diagnosis of simple bone cysts was clear based on related images, especially in X-rays and MRI, no preoperative biopsy was performed. Moreover, the clinical and radiologic features were used for differential diagnosis of SBC from another cystic lesions based on a previous report [3], including aneurysmal bone cyst, fibrous dysplasia, enchondroma, eosinophlic granuloma and intraosseous ganglia. Briefly, aneurysmal bone cyst on roentgenograms appear as a lytic, eccentric, intramedullary bone lesion, with a transverse diameter that is wider than the epiphyseal plate, and the MRI images of these lesions show double-density fluid levels and septations. Fibrous dysplasia cases can be distinguished by ground glass appearance of the matrix. Enchondromas are distinct radiolucent intramedullary lesions with thinning and expansion of the cortices, which are usually happened in short tubular bones of the hands and feet. Eosinphilic granuloma frequently involves axial skeleton than appendicular skeleton, while intraosseous ganglia are small radiolucent lesions that mainly observed in the epiphysis and subchondral region.

The demographic data of this study group are summarized in Table 1. Eleven males and 7 females with an average age of 10.94 years (range, 7 to 15 years) were included in this study, and the mean follow-up period was 40 months (range, 19–65 months). Most of the cysts were located in the metaphyseal, isolated diaphyseal or metaphyseal-diaphyseal regions of the humerus. According to the standard proposed by Neer et al. [27], the cysts were distinguished into four grades, and the classification was based on the severity of the lesion. As a result, those cysts were found active in 16 cases and inactive in 2 cases. Most of the patients were brought to the outpatient by their parents due to upper arm pain or accidental injury, or diagnosed pathological fracture in other institutions. A pathological fracture happened in 12 cases. Recurrence, partial healing and pathological fracture were all our surgical indications.

**Table 1:** Demographic data and general data for study

ASE	L or R	Frature or not	Stage	Treatment	Follow-up	Radiographic healing	Complica-tions	VAS (pre)	VAS (post)
1	L	Y	active	S + EIMN	19	grade 1	N/A	6	1
2	R	Y	active	S + EIMN	24	grade 1	N/A	4	0
3	R	N	active	S + EIMN	28	grade 1	N/A	2	0
4	L	Y	active	S + EIMN	37	grade 1	N/A	4	0
5	R	N	active	S + EIMN	42	grade 1	N/A	2	0
6	R	Y	inactive	S + EIMN	31	grade 1	N/A	5	0
7	L	Y	active	S + EIMN	53	grade 1	N/A	6	1
8	L	Y	active	S + EIMN	34	grade 2	N/A	5	1
9	R	N	active	S + EIMN	48	grade 1	irritation	3	0
10	L	Y	active	S + EIMN	60	grade 1	N/A	5	1
11	R	N	active	S + EIMN	44	grade 1	N/A	3	0
12	L	Y	active	S + EIMN	31	grade 2	N/A	6	0
13	R	Y	active	S + EIMN	55	grade 1	valgus deformity	6	1
14	L	Y	active	S + EIMN	65	grade 2	N/A	5	0
15	L	Y	active	S + EIMN	58	grade 1	N/A	5	0
16	R	N	inactive	S + EIMN	39	grade 2	N/A	3	0
17	R	N	active	S + EIMN	25	grade 1	N/A	2	1
18	R	Y	active	S + EIMN	29	grade 1	N/A	6	0

(*N/A* not complications)

### Surgical technique

After a review of related imaging studies, according to symptoms and physical signs, a conclusion that a benign tumor was the more likely diagnosis was drawn. Surgery was always performed under general anesthesia and radiographic control, and it started from an incisional penetration with a big syringe in the region of the bone cyst located under a C-arm X-ray. The order of the penetration was from the distal part of the cast to the proximal and the surgeon should try to avoid touching vital nerves and vessels in case of hurting any of them. The syringe was through minimally percutaneous penetration, trying to avoid open incision. If the cystic cavity was too large, two or three penetrative points were necessary. Then extracted the cyst liquid which was yellow and transparent, and hemorrhagic combined with pathological fractures. We extracted the fluid in the cyst with a 5 ml medical syringe and send it to histopathologic examination. Afterwards, wash the cavity with normal saline and cause no further damage to the wall.

The fluid in the cyst was centrifuged, smeared onto a slide, evaluated by H&E staining and observed under microscope (40 ×, 100 × and 200 ×). Patients were diagnosed based on their symptoms as well as the results of X-ray, CT, MRI, and pathology.

Titanium elastic intramedullary nailing (TEN) was applied, which meant to insert elastic intramedullary nails through windows cut on the lateral cortex of the distal of humerus. There were two operative approaches. One was to operate on the medial epicondyle of the humerus and ectepicondyle of humerus, and the other was to operate on the same side of the lateral of ectepicondyle of humerus. The surgeon should be careful not to cause any ulnar nerve injure. For the patients with pathological fractures, reduction should first be performed to reduce injury. The length of the nails was variably selected according to the patient’s sex, age, and the bone length (confirmed on the basis of the preoperative images). The diameter of the nails was selected according to the criterion which said 2 nails would occupy 2/3 of the minimum diameter of the medullary cavity, and the longest one was not allowed to be beyond the epiphyseal plate line. In case of disturbing epiphyseal growth, the distal end of the nails was left in a manner to avoid irritation of the surrounding soft issues. The procedure was under the guidance of a C-arm system. As the elastic intramedullary nail passed through the cyst, decompression and drainage were completed.

Methylprednisolone acetate was injected into the cavity through previous percutaneous penetrative point at a variable dose according to the volume of the cavity. 200 to 2000 mg of methylprednisolone acetate (40 mg/ml) was injected into the humeral cavity. Since the elastic intramedullary nail had passed through the bone cyst and the decompression was done, the internal drainage was accomplished.

### Postoperative patient management

All the patients wore a sling after operation, the patient was checked every 2 days. On average, it took about 7–14 days for them to stay in hospital. Active finger and waist motion, and passive elbow and shoulder motion were allowed immediately after operation. Active elbow and passive shoulder motion were allowed 4 weeks after operation. Active shoulder motion was allowed 6–8 weeks after operation.

### Removal of the intramedullary nailing

For the well-healed SBC patients, the elastic intramedullary nails were removed as soon as possible. As the protocol to remove the lastic intramedullary nail in the treatment of children humerus fracture, we expanded the original incision to expose the elastic intramedullary nails while protecting the protect local soft tissue, blood vessels and nerves. Then clenched the end of the elastic intramedullary nails with pliers, knocked gently along the long axis of the humerus longitudinal direction to loss the nails, and pulled out elastic intramedullary nail in the opposite direction. For the nails that unable to observed accurately, we would place a 5 ml syringe needle nearby, and search for the nail by intraoperative fluoroscopy (C-arm), remove part of the bone cortex to expose the nail, and then remove it as described above.

### Radiological and functional analysis

Radiological and functional follow-ups were mostly taken in the orthopedic outpatient clinic, and patients’ radiographs were evaluated at admission (preoperative), 1 week, 1 month, 2 months, 3 months, 6 months, 12 months and 24 months after the operation, as well as the last follow up. All the patients were asked to take an anteroposterior and lateral radiograph of the humerus.

Musculoskeletal Tumor Society (MSTS) criteria was used to assess the function [28] before and after the operation (Table 2). Using this scoring system, each patient’s emotion, function and pain were evaluated, besides, weight lifting for upper lesions, hand position and hand skills were also recorded (Table 3).

**Table 2:** MSTS (preoperation and postoperation)

ASE	pre-	post 3 months	post 6 months	post 12 months	post 24 months	last follow-up
1	10	22	26	28	0	29
2	8	24	27	29	0	30
3	12	23	27	28	29	29
4	10	26	28	29	30	30
5	9	21	26	28	30	30
6	11	23	25	29	29	29
7	12	25	28	29	29	29
8	8	23	27	29	30	30
9	13	25	28	29	29	29
10	11	26	28	29	30	30
11	12	24	26	27	27	27
12	9	23	26	28	29	29
13	10	22	28	29	30	30
14	14	26	27	29	30	30
15	12	24	27	28	29	29
16	10	23	25	27	28	28
17	9	24	26	28	29	29
18	12	25	27	29	30	30

**Table 3:** MSTS ranking score

	MSTS ranking score
Perfect	23,above23
Good	15–22
Moderate	8–14
Bad	8,below8

Treatment success was evaluated by Capanna criteria [29], which includes four grades, grade 1: complete healing, fully filled with bone; grade 2: partial healing with a small residual cystic area remains; grade3: partial healing with a large residual cystic area remains; grade 4: partial healing, with response (Table 4). In order to make statistical analysis easier, we made a slight revision of Capanna criteria. As shown in Table 5, we defined grade 1 to be 4 points, grade 2 to be 3 points, grade 3 to be 2 points, and grade 4 to be 1 point. Preoperative and postoperative results of the last follow-up were recorded in Table 6.

**Table 4:** Capanna criteria

Classification	Clinical manifestation
Grade 1	Completely healing, fully filled with bone
Grade 2	Partially healing, with a small residual cystic area remains
Grade 3	Partially healing, with a large residual cystic area remains
Grade4	Partially healing, with response

**Table 5:** modified Capanna criteria

Classification	Capanna ranking score
Grade1	4
Grade2	3
Grade3	2
Grade4	1

**Table 6:** Clinical detail of surgical groups

	Capanna		VAS	
	pre	post	pre	post
Average ± SD	1 ± 0	3.777 ± 0.427	4.33 ± 1.49	0.33 ± 0.48

### Complications

Early or late complications were recorded, including wound problems, infection, refracture, deformity and nerve injury.

### Statistical analysis

Statistics work was done with SPSS17.0 statistical software (USA). Paired t tests were used to compare the MSTS scores, the visual analog scale (VAS) scores and the Capanna scores separately before and after operation. The statistically significant difference level was set at * *p* < 0.05, **p < 0.01 and ***p < 0.001.

## Results

**Fig. 1:**
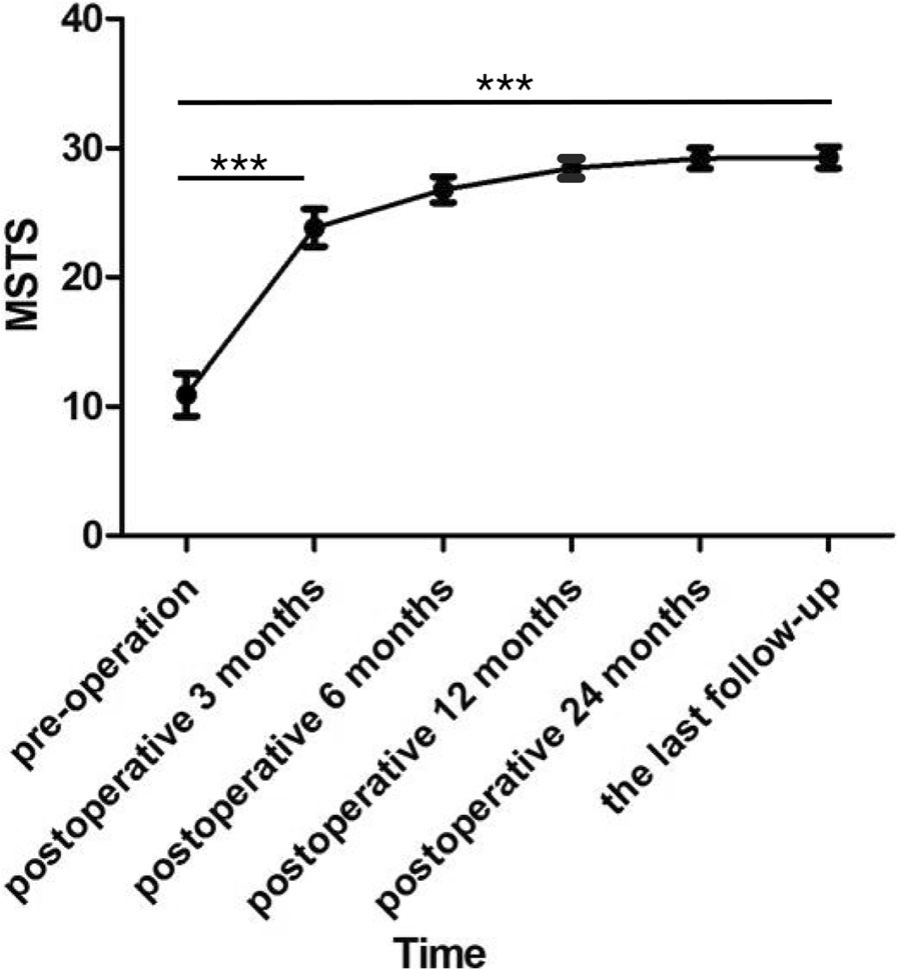
The scores of MSTS. The graph shows the preoperative and postoperative follow-up MSTS scores. Functional scores increased progressively until the end of postoperative 12 months, and then a plateau was reached and preserved throughout the rest of follow-up. There were significant difference between the MSTS scores of pre-operation and postoperative 3 months groups (*P* = 2.36277E-17) as well as those of postoperative 3 months and postoperative 24 months groups (*P* = 1.28483E-09). ***Significant difference, *P* < 0.001

**Fig. 2:**
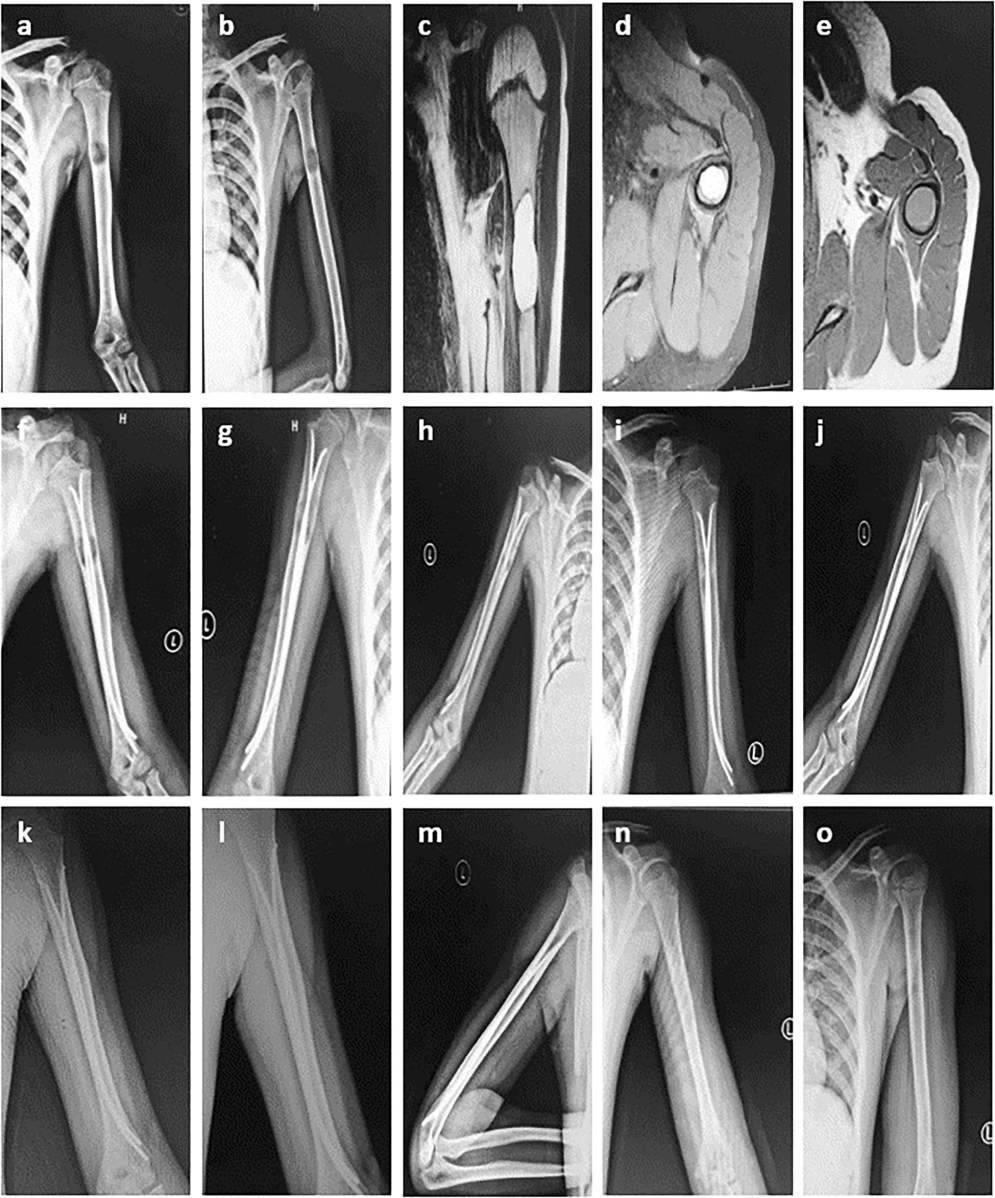
Radiographs of a 10-year-old boy who presented with pathological fracture of the left humerus. **a**-**b** Anteroposterior and lateral radiograph on admission. **c**-**e** MRI indicates a low signal on T1-weighted images and a homogeneous high signal on T2 weighting. **f**-**k** After the operation of minimally invasive intramedullary decompression and drainage with ESIN combined with intralesional injections of steroids; At the interval points of postoperative 1 weeks, 2 months, 6 months, 9 months, 12 months, 14 months, the lesion is significantly smaller and shows signs of healing gradually. At 14 months, Radiograph show complete cyst healing (Capanna grade 1 healing). **l**-**o** At 16 months, the bone cyst has resolved, and then the intramedullary nails are removed. **n**-**o** Radiographs show complete cyst healing (Capanna grade 1 healing) after second surgery

**Fig. 3:**
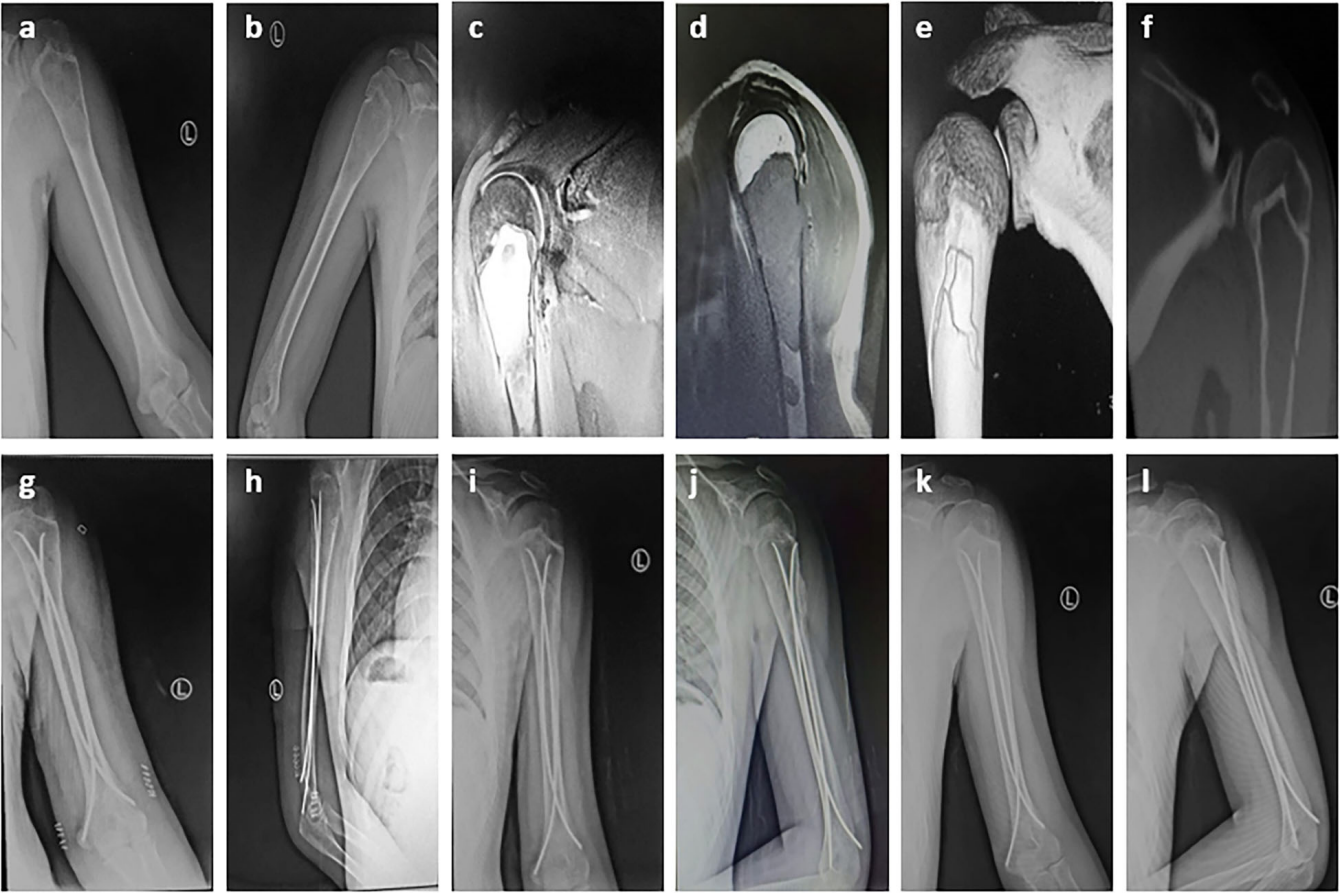
Radiographs of an 11-year-old boy who presented with pathological fracture of the left humerus. **a**-**b** Anteroposterior and lateral radiograph on admission. **c**-**d** MRI indicates a low signal on T1-weighted images and a homogeneous high signal on T2 weighting. **e**-**f** Three-dimensional CT image reconstruction and a sagittal CT scanning show fracture of proximal humeral bone cyst. **g**-**l** After the operation of minimally invasive intramedullary decompression and drainage with ESIN combined with intralesional injections of steroids; At the interval points of postoperative 1 weeks, 2 months, 6 months, the lesion is significantly smaller and shows signs of healing gradually, the fracture gradually healed, Radiographs show complete cyst healing (Capanna grade 1 healing)

All the patients were given continued follow up for 19 to 65 months. The ESIN were able to be removed within 1–2 years postoperatively. In this study, the average time of the surgery to remove ESIN was 14.5 months after the first operation. After the ESIN was removed, the follow-up continued for 25.61 ± 13.57 months, and no reoccurs was happed in our cases (Table 7). One patient was lost to follow-up 7 months after removing the nail, but he had been well healed at that time. The MSTS scores significantly increased from 3 months to 24 months after the operation (*P* = 1.28483E-09), and the most significant change happened within 3 months after the operation compared to the preoperative status (*P* = 2.36277E-17). And there were no significant differences during the period from 24 months after operation to the last follow-up (*P* = 0.16) (Figs. 1, 2 and 3). As shown in Table 6, the modified Capanna criteria scores showed that the complete healing happened in 14 cases (78%, 14/18), while partial healing was founded in 4 cases (22%, 4/18). The average Capanna score was 1 ± 0 before and 3.777 ± 0.427 after operation (*P* < 0.05). The treatment regimen used in this study was effective in all cases**.** All patients had pain relief. The average VAS score on admission was 4.33 ± 1.49, which decreased to 0.33 ± 0.48 after operation, and there was a significant statistical difference with *P* < 0.05. No nonunion occurred in all the combined pathological fracture patients who suffered from displaced fractures or microfractures. However, the healing time varies.

**Table 7:** Follow-up after the first treatment and ESIN removal

ASE	Total follow-up duration (months)	Time of ESIN removal (months)	Follow-up duration after ESIN removal (months)
1	19	12	7
2	24	12	12
3	28	13	15
4	37	16	21
5	42	19	23
6	31	16	15
7	53	25	28
8	34	18	16
9	48	7	41
10	60	17	43
11	44	16	28
12	31	10	21
13	55	12	43
14	65	13	52
15	58	14	44
16	39	12	27
17	25	13	12
18	29	16	13
Mean ± SD	40.11 ± 13.77	14.5 ± 3.96	25.61 ± 13.57

The function of the elbow and shoulder obtained complete recovery with the fixation of elastic intramedullary nails, and the average recovery time was found to be 9 weeks (range: 8-11 weeks). Besides, the elbow and shoulder were allowed to exercise 3 weeks postoperatively. The full weight of the humerus was permitted when the x-ray show signs of bone union, which was usually 6 months postoperatively. In only one case, the elastic intramedullary nails were manipulated under general anesthesia after the 7 months because of the patient’s skin irritability induced by intramedullary nails loosening.

**Fig. 4:**
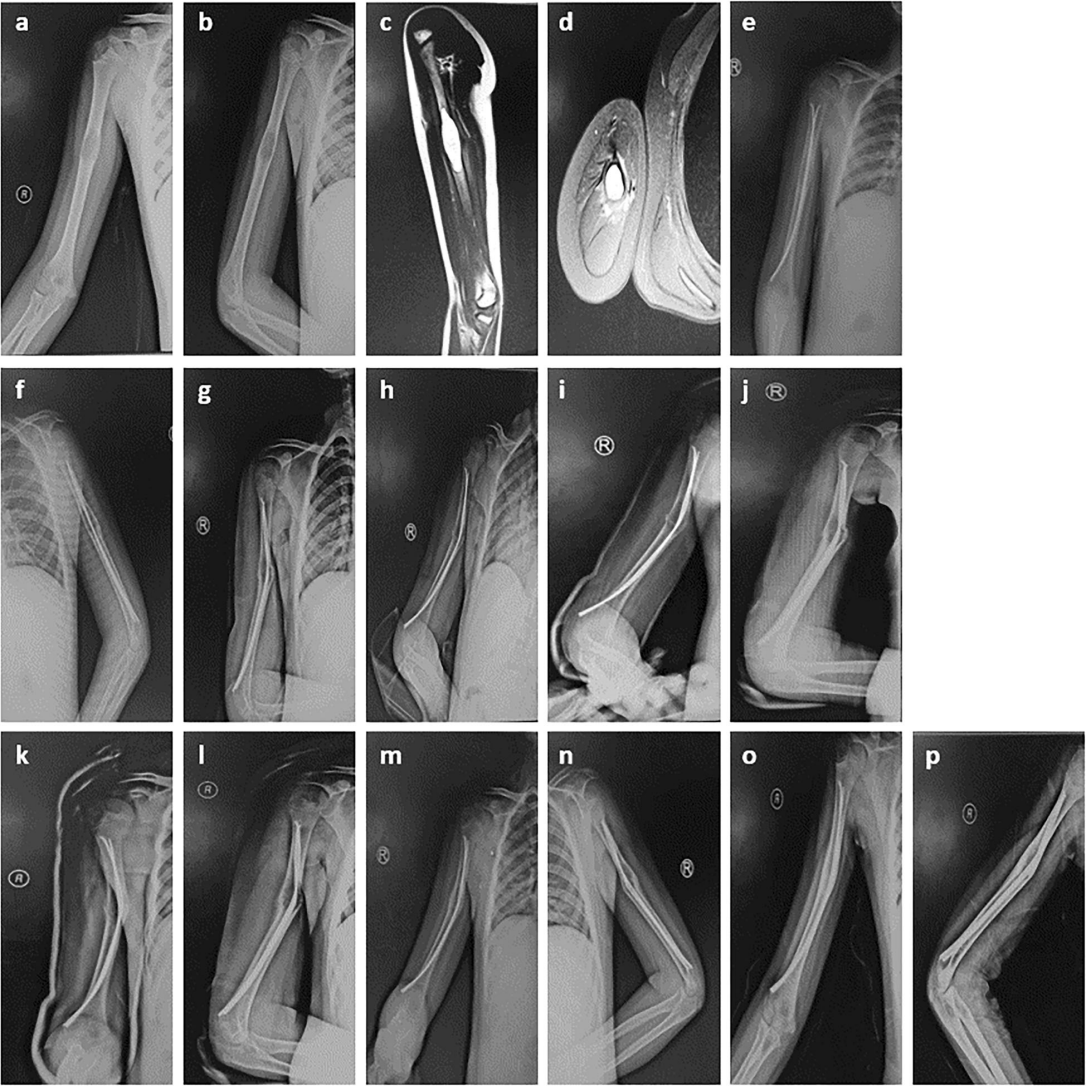
Radiographs of a 10-year-old boy who presented with pathological fracture of the right humerus. **a**-**b** Anteroposterior and lateral radiograph on admission. **c**-**d** MRI indicates a big bone cyst in humerus. **e**-**f** After the operation, postoperative 1 weeks Radiographs show a good position of the fixation. **g**-**h** At 1 month after surgery, an accident is happened, posteroanterior X-ray shows a valgus deformity and a secondary fracture in the cyst region. **i**-**j** An”O″ shaped cast fixation was done after reduction in outpatient clinic, X-ray shows a valgus deformity. **k**-**n** At the interval points of postoperative 6 weeks, 2 months, Radiographs show a valgus deformity. **o**-**p** Radiographs show complete cyst healing while there is a valgus deformity, although the position of photograph was not satisfactory, there was no functional and visual defects

The complications of the surgical method were summarized in Table 8. There was one case pf refracture caused by accident in 1 month after surgery, and the valgus deformity was showed by posteroanterior X-ray in Fig. 4. However, the parents of this child refused a second surgery, and an “O” shaped cast fixation was done after reexamination in outpatient clinic. The child was treated with conservative treatment including the use of a sling for 4 weeks. At follow-up, although the radiographic result was not satisfactory, there was no functional and visual defects, and no nerve injury.

**Table 8:** complications

Complications	Cases
Wound	1
Infection	0
Refracture	0
Deformity	1
Nerve injury	0
Others	0

## Discussion

Many researchers believed that SBCs did not require special treatment, and it could be resolved before the bone matured. A considerable of people think that a fracture might cure the cyst, however, spontaneous healing occurs only about 5–10% of all cases [30, 31]. Besides, risk factors such as pain, refracture and deformity had baffled parents and doctors [32, 33]. The main goals when treating SBCs are tantamount to decrease the risk of pathological fracture, assist cyst healing and stop pain. So, exploring a safe, effective, minimally invasive treatment and quick recovery methods had always been the pursuit. Various treatments for SBCs have been development, but there is no agreement on the best strategy. Open surgical methods, including curettage and bone grafting were regarded as the most common procedures methods [27]. But the elastic intramedullary nailing changes the above protocol. Firstly, it can provide mechanical stability for effective fixation. Secondly, it is a minimally invasive surgery, which can protect the blood supply of the bone. Erol et al. reported a comparative study for the treatment of simple bone cysts of the humerus, which suggested that although perfect functional results were possible with open curettage and grafting, continuous intramedullary decompression with elastic nails led to a higher radiographic healing rate [34]. Meanwhile, Erol’s group also proved that, the treatment with intramedullary nail was able to support the restore of bone integrity by allowing early motion as well as preventing refracture and subsequent deformity in the majority of patients [19].

Percutaneous and less invasive methods are widely promoted because of the advantages of lower infection rates, fewer wound problems, smaller scars, less anesthetic and fewer complications, making it easier for patients and their families to accept a surgery. The most common ones are percutaneous needle aspiration [35] and injection treatment of steroid or autologous bone marrow [14, 36]. A study compared treatments in which the simple bone cysts were injected with bone marrow or steroid [12]. Two years after treatment, X-ray examination showed that successful healing of bone cysts was more common in children who had received steroid injections. But the difference is small. Steroid therapy has many advantages, such as its simplicity, low cost, high availability and lack of direct post-operative adverse effects, etc. However, one of the most common problems with steroid therapy in SBCs is its long duration.

Many researchers had demonstrated the application of ESIN in the treatment of SBCs [37, 38]. The intramedullary nails can make a balance between the pressure of the cyst and the medullary cavity, relieve the venous obstruction and improve the local blood circulation. Bumci [39] et al. considered that a cut-through between the medullary canal and the cyst cavity could decrease the pressure, improve the microcirculation between them and stimulate bone formation. Besides, the curved and flatted nail tip can effectively damage the cystic wall, and make it easier for ossification and formation of an osteogenic microenvironment in the cystic cavity. Most importantly, elastic intramedullary nailing provides mechanical stability for the prevention or effective fixation of pathological fractures, because it is difficult and sometimes isolating to limit a child for a considerable period of time from activities which might fracture the humerus.

Through reviewing the etiology and pathology of the SBCs, the most commonly accepted hypotheses include elevated intraosseous pressure due to venous obstruction and bone destruction caused by PGE2 and IL-1β enzymes [9, 40]. On the basis of existing hypothesis, we can conclude a combination therapy that can not only achieve effective decompression but also inhibit the obstruction of PGE2 and IL-1β enzymes by applying both percutaneous needle aspiration and injection steroid. Moreover, to shorten the treatment duration and provide early mechanical stability for the SBCs with pathological fractures while promoting bone healing, we combined application of elastic intramedullary nails (ESIN) with those two methods. To our knowledge, no such treatment was reported previously. On the basis of the previous work, we combined elastic intramedullary nails with methylprednisolone acetate. In this study, all the children got a X ray, then an MRI scan for diagnosis. No preoperatively biopsy was given. After treatment with the combination therapy, 100% complete or partial radiographic healing was achieved in the 18 patients with no recurrence or adverse response, and no patient suffered a second surgery to eradicate the cyst. This fully demonstrated the advantages and development prospects of this combination therapy. However, as one patient’s intramedullary nail had loosened and the patient developed skin irritability, an earlier removal surgery was manipulated under general anesthesia.

It is still controversial about when to remove the internal nail. Some surgeons prefer to leave the nails till the children are outgrown or after puberty. It is based on the worry about the higher tendency to get recurrent cysts in relative younger patients. However, this will increase the difficulty to remove the elastic intramedullary nail. It is usually buried into bones after several years. The other surgeons like to take it out earlier. Although it may increase the recurrent cysts, it is easy to remove. In our study, we removed all the nails 1–2 years postoperatively, and there was no recurrent cyst. But because of our small sample and short follow-up time, we are still not able to tell which method was better. It needed to be confirmed in future big sample, multicenter and prospective study. Moreover, there were some other limitations in this study: Firstly, the study population was small and a large number of samples were necessary to draw more reliable conclusions. Secondly, the amount of injections of steroids was not the same.

## Conclusion

In our study, minimally invasive intramedullary decompression and drainage with ESIN and intralesional injections of steroids had achieved perfect results in the treatment of SBCs. In the future study, we should expand the sample size, optimize the dose of steroid, further explore the mechanism of this method in the treatment of SBCs and apply it in the treatment of other diseases.

## Data Availability

The datasets during and/or analyzed during the current study are availablefrom the corresponding author on reasonable request.

## Abbreviations

CT: Computed tomography
ESIN: Elastic stable intramedullary nailing
IL-1: Interleukin-1
MRI: Magnetic resonance imaging
MSTS: Musculoskeletal Tumor Society
PGE2: Prostaglandin E2
SBC: Simple bone cyst
TEN: Titanium elastic intramedullary nailing
UBC: Unicameral bone cyst
VAS: Visual analog scale
